# Chimpanzee accumulative stone throwing

**DOI:** 10.1038/srep22219

**Published:** 2016-02-29

**Authors:** Hjalmar S. Kühl, Ammie K. Kalan, Mimi Arandjelovic, Floris Aubert, Lucy D’Auvergne, Annemarie Goedmakers, Sorrel Jones, Laura Kehoe, Sebastien Regnaut, Alexander Tickle, Els Ton, Joost van Schijndel, Ekwoge E. Abwe, Samuel Angedakin, Anthony Agbor, Emmanuel Ayuk Ayimisin, Emma Bailey, Mattia Bessone, Matthieu Bonnet, Gregory Brazolla, Valentine Ebua Buh, Rebecca Chancellor, Chloe Cipoletta, Heather Cohen, Katherine Corogenes, Charlotte Coupland, Bryan Curran, Tobias Deschner, Karsten Dierks, Paula Dieguez, Emmanuel Dilambaka, Orume Diotoh, Dervla Dowd, Andrew Dunn, Henk Eshuis, Rumen Fernandez, Yisa Ginath, John Hart, Daniela Hedwig, Martijn Ter Heegde, Thurston Cleveland Hicks, Inaoyom Imong, Kathryn J. Jeffery, Jessica Junker, Parag Kadam, Mohamed Kambi, Ivonne Kienast, Deo Kujirakwinja, Kevin Langergraber, Vincent Lapeyre, Juan Lapuente, Kevin Lee, Vera Leinert, Amelia Meier, Giovanna Maretti, Sergio Marrocoli, Tanyi Julius Mbi, Vianet Mihindou, Yasmin Moebius, David Morgan, Bethan Morgan, Felix Mulindahabi, Mizuki Murai, Protais Niyigabae, Emma Normand, Nicolas Ntare, Lucy Jayne Ormsby, Alex Piel, Jill Pruetz, Aaron Rundus, Crickette Sanz, Volker Sommer, Fiona Stewart, Nikki Tagg, Hilde Vanleeuwe, Virginie Vergnes, Jacob Willie, Roman M. Wittig, Klaus Zuberbuehler, Christophe Boesch

**Affiliations:** 1 Max Planck Institute for Evolutionary Anthropology (MPI EVAN), Deutscher Platz 6, 04103 Leipzig, Germany; 2 German Centre for Integrative Biodiversity Research (iDiv) Halle-Leipzig-Jena, Deutscher Platz 5e, 04103 Leipzig, Germany; 3 Wild Chimpanzee Foundation (WCF), Deutscher Platz 6, 04103 Leipzig, Germany; 4 Chimbo Foundation, Amstel 49, 1011 PW Amsterdam, Netherlands; 5 Ebo Forest Research Project, BP3055, Messa, Cameroon; 6 The Aspinall Foundation, Port Lympne Wild Animal Park, Hythe, Kent, UK; 7 West Chester University, Departments of Anthropology & Sociology and Psychology, West Chester, PA, USA; 8 Wildlife Conservation Society (WCS), 2300 Southern Boulevard. Bronx, New York 10460, USA; 9 Korup Rainforest Conservation Society, c/o Korup National Park, P.O. Box 36 Mundemba, South West Region, Cameroon; 10 Lukuru Foundation, 1235 Avenue des Poids Lourds/Quartier de Kingabois, Kinshasa, DRC; 11 WWF Cameroon Country Office, BP6776; Yaoundé, Cameroon; 12 Agence National des Parcs Nationaux (ANPN) Batterie 4, BP20379, Libreville, Gabon; 13 School of Natural Sciences, University of Stirling, Stirling, UK; 14 Institute de Recherche en Ecologie Tropicale, Libreville, Gabon; 15 University of Cambridge, Pembroke Street, Cambridge, UK CB2 3QG; 16 Arizona State University, PO Box 872402, Tempe, AZ 85287-2402 USA; 17 Lester E. Fisher Center for the Study and Conservation of Apes, Lincoln Park Zoo, 2001 North Clark Street, Chicago, Illinois 60614 USA; 18 Institute for Conservation Research, Zoological Society of San Diego, Escondido, CA 92025, USA; 19 School of Natural Sciences and Psychology, Liverpool John Moores University, James Parsons Building, Rm653 Byrom Street, Liverpool L3 3AF, UK; 20 Iowa State University, Department of Anthropology, 324 Curtiss Hall, Ames, IA 50011, USA; 21 West Chester University, Department of Psychology, 700 S High St., West Chester, PA, 19382 USA; 22 Washington University Saint Louis, Department of Anthropology, One Brookings Drive, St. Louis, MO 63130, USA; 23 University College London, Department of Anthropology, 14 Taviton Street, London WC1H 0BW, UK; 24 KMDA, Centre for Research and Conservation, Royal Zoological Society of Antwerp, Koningin Astridplein 20-26, B-2018 Antwerp, Belgium; 25 Taï Chimpanzee Project, Centre Suisse de Recherches Scientifiques, BP 1301, Abidjan 01, Côte d’Ivoire; 26 Université de Neuchâtel, Institut de Biologie, Rue Emile-Argand 11, 2000 Neuchâtel, Switzerland

## Abstract

The study of the archaeological remains of fossil hominins must rely on reconstructions to elucidate the behaviour that may have resulted in particular stone tools and their accumulation. Comparatively, stone tool use among living primates has illuminated behaviours that are also amenable to archaeological examination, permitting direct observations of the behaviour leading to artefacts and their assemblages to be incorporated. Here, we describe newly discovered stone tool-use behaviour and stone accumulation sites in wild chimpanzees reminiscent of human cairns. In addition to data from 17 mid- to long-term chimpanzee research sites, we sampled a further 34 *Pan troglodytes* communities. We found four populations in West Africa where chimpanzees habitually bang and throw rocks against trees, or toss them into tree cavities, resulting in conspicuous stone accumulations at these sites. This represents the first record of repeated observations of individual chimpanzees exhibiting stone tool use for a purpose other than extractive foraging at what appear to be targeted trees. The ritualized behavioural display and collection of artefacts at particular locations observed in chimpanzee accumulative stone throwing may have implications for the inferences that can be drawn from archaeological stone assemblages and the origins of ritual sites.

In both contemporary and ancient human societies, stone piles are often used to mark natural cavities in the landscape for caching food, as well as paths and important places^1^, and can hold a more symbolic meaning for burials^2^, ceremonial counting^3^, and the establishment of shrines^4^. Through archaeology, analyses of stone assemblages have provided us with insight into the technological and cognitive abilities of ancestral hominins^5^. It is therefore notable that the use of stone tools has also been observed in wild populations of nonhuman primates, including chimpanzees, one of our closest living relatives^6^,^7^. Primate archaeology has therefore emerged as a new field of research where archaeological evidence from nonhuman primates can be compared to our own^8^,^9^. Any similarities may not only challenge, but may also illuminate the interpretations of stone accumulations in human prehistory.

Along with chimpanzees^7^, bearded capuchin monkeys in Brazil^10^ and long-tailed macaques in Thailand^11^ are also known to use stone hammers to crack open food encased by an outer shell. The use of stone tools in an extractive foraging context provides individuals with an immediately observable benefit, unlike other forms of stone tool use observed in nonhuman primates, such as stone throwing. Stone throwing was first described by Goodall who documented aimed throwing of sticks and rocks by male chimpanzees during agonistic displays^12^ and was later described for other nonhuman primates, particularly captive Japanese macaques^13^, wild baboons^14^ and capuchins^15^. Bearded capuchins have also been documented to bang stones in the wild, presumably to deter predators^16^, and females of this same species have been observed to throw stones during courtship contexts^15^. Nonetheless, previous examples of nonhuman primates throwing stones in the wild were limited to a single group^15^ or anecdotal observations^12^,^14^. As such, stone throwing and banging has not been described as a customary behaviour of any nonhuman primate species. Stone handling, however, is a behavioural tradition found uniquely among Japanese macaques and has been observed in multiple groups^13^,^17^. Although stone handling is not a form of tool use^18^, the behaviour occurs frequently enough to result in recognizable use-wear patterns^19^. Of particular interest is the accumulation of stones at ‘play stations’ which has the potential to leave behind archaeological evidence of the behaviour^19^, similar to chimpanzee and capuchin nut-cracking sites^8^.

Chimpanzees exhibit the greatest variation in tool-use behaviours of any animal, second only to humans^7^,^18^. While all studied chimpanzee populations utilize leaves for the acquisition of food, some additionally use sticks, twigs, and even spears^7^,^18^,^20^. The majority of stone tool use has been observed in Western chimpanzee communities, where stone hammers and anvils are used to crack open nuts^20^,^21^, and stone cleavers are used to cut up large *Treculia* fruits^22^. The diversity and variation in the behavioural repertoire of chimpanzee populations across Africa strongly supports group-specific and socially-learned cultural traditions in our closest living relatives^7^,^20^. However, our current knowledge about the repertoire of chimpanzee tool use and inferred cultural transmission stems from a very limited number of long-term field sites, patchily distributed across the entire range of wild chimpanzees in Africa^20^. This limitation becomes particularly apparent when compared to the large number of human populations studied for similar questions on the evolution of culture^7^. The regular discoveries of additional tool-use behaviours in previously unstudied chimpanzee populations, or variants of behaviours already described, suggest that the true spectrum of natural chimpanzee behaviour is likely to be much broader than what is currently known^7^.

In an effort to overcome this limitation, we sampled a large number of previously unstudied and/or unhabituated chimpanzee groups across the species’ range as part of the Pan African Programme: The Cultured Chimpanzee (henceforth “PanAf”)^23^, to study the influence of ecological conditions on the observed spatial variation in tool-use behaviours and inferred cultural patterns. At each PanAf research site, a broad spectrum of non-invasive sampling methods were applied for a 14–17 months period (see Supplementary Table 1), according to a standard protocol^24^. Here we present the first evidence of a previously undocumented, chimpanzee stone tool-use behaviour arising from the data collection of the PanAf.

## Results

**Figure 1:**
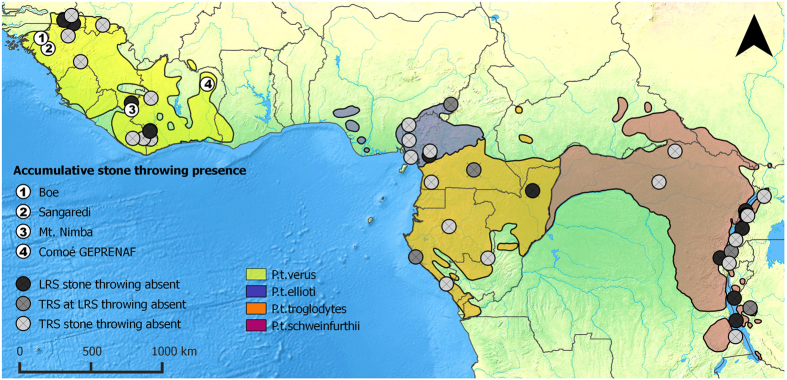
Chimpanzee range map showing the geographic distribution of accumulative stone throwing populations. The map shows the locations of all chimpanzee populations studied across Africa including the four PanAf temporary research sites (TRSs) where accumulative stone throwing behaviour was observed (*white circles*; 1: Boé, Guinea-Bissau; 2: Sangaredi, Guinea; 3: Mt. Nimba, Liberia; 4: Comoé GEPRENAF, Côte d’Ivoire). Chimpanzee accumulative stone throwing was not observed at all other research sites: PanAf TRS (*light grey circles*), PanAf TRS carried out at mid- to long-term chimpanzee research sites (*dark grey circles*) and PanAf TRS carried out at mid- to long-term research sites of habituated chimpanzees (*black* *circles*). See also Supplementary Table 1. (Map created by M. Arandjelovic using QGIS version 2.6.1: http://www.qgis.org/en/site/).

Prior to our study, there were reports and anecdotes of wild chimpanzees throwing and banging stones in Liberia, Guinea, and Guinea-Bissau (A.G. pers. obs.) in addition to occasional observations of habituated chimpanzees throwing rocks and other objects during male threat displays^12^. The first direct observations of stone throwing behaviour in association with the presence of accumulated stones at specific trees (henceforth “accumulative stone throwing”) were recorded by camera traps on March 24, 2011, at the Sangaredi PanAf temporary research site (TRS) in Guinea (Fig. 1; TRS #2). Following this observation, additional data collection procedures particular to this behaviour were incorporated into the PanAf protocol and administered to all TRSs across Africa to ensure comparable data across research sites (see Methods).

### Chimpanzee accumulative stone throwing behaviour

**Figure 2:**
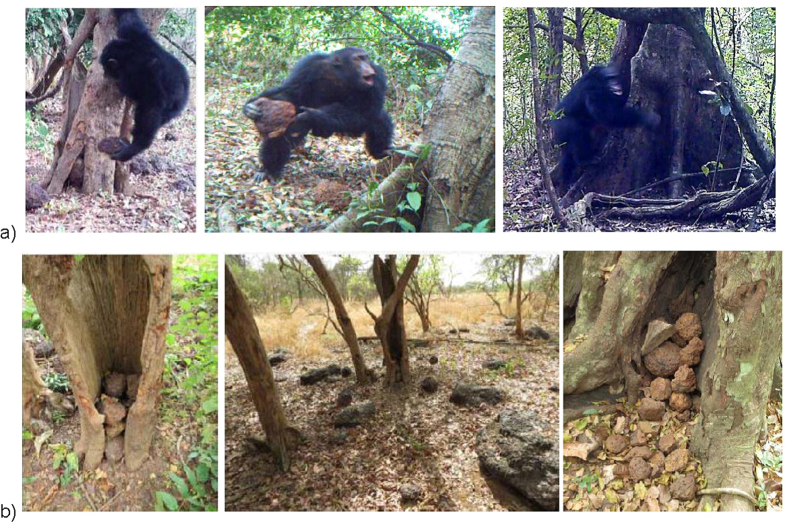
Photographs and stills of accumulative stone throwing behaviour and sites. (**a)** Adult male chimpanzee tossing a stone; hurling a stone (Boé, Guinea-Bissau); and banging a stone (Comoé GEPRENAF, Côte d’Ivoire). (**b)** Boé, Guinea-Bissau landscape: stones accumulated in a hollow tree; a chimpanzee accumulative stone throwing site; and stones accumulated in-between buttress roots (see also Supplementary Movies 1–7).

**Figure 3:**
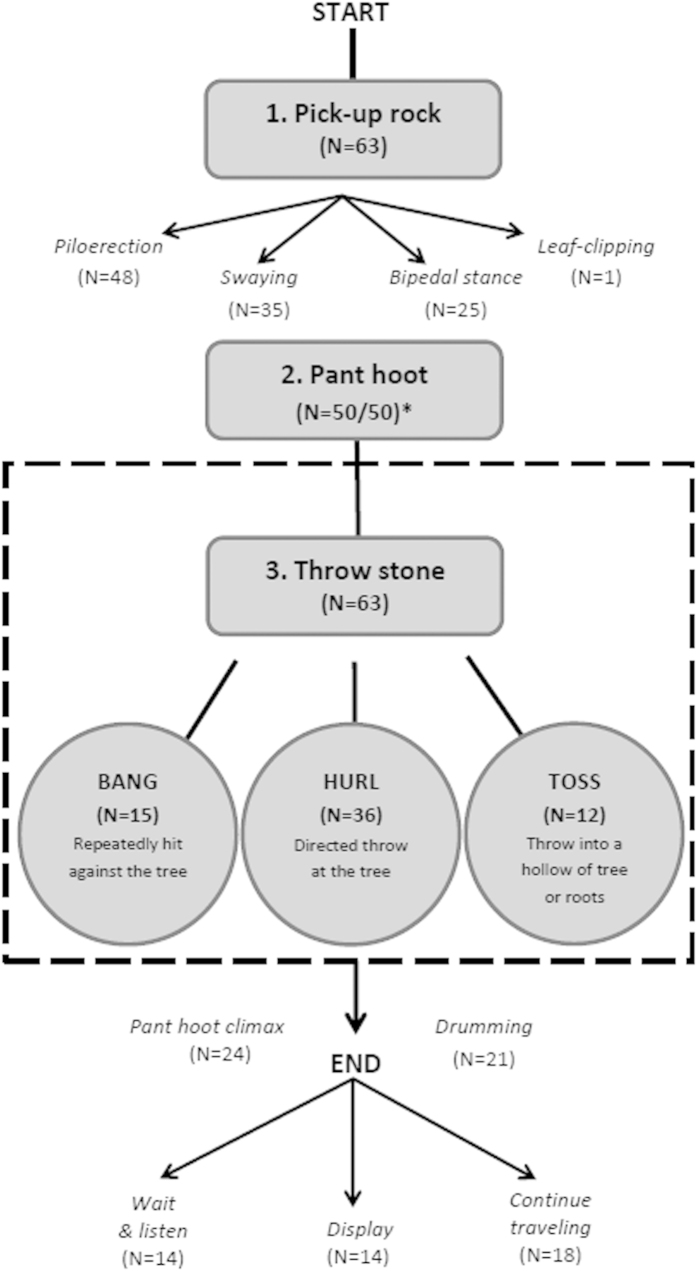
Flow chart describing the behavioural elements observed in chimpanzee accumulative stone throwing. Three key behaviours were common to all observations of adult (N = 63) chimpanzee accumulative stone throwing (*grey rectangles*): 1) picking-up and handling a rock, 2) pant hoot introduction and/or build up phase, and 3) throwing the stone. Other behaviours were only sometimes observed or were observed in combination with one another (*italicized*). *Only 50 videos contained audio, all of which recorded a pant hoot vocalization.

Thirty-one TRSs located within the *Pan troglodytes* range were sampled between 2011 and 2015 for a period of 14–17 months. An additional three TRSs were on-going and studied for less than 14 months, for a total of 34 (see Supplementary Table 1). At four TRSs: (Boé, Guinea-Bissau; Sangaredi, Guinea; Mt. Nimba, Liberia and Comoé GEPRENAF, Côte d’Ivoire; Fig. 1) we found multiple hollow and/or buttressed trees exhibiting clear signs of wear with an accumulation of rocks at their base or inside the tree (Fig. 2). Using remote video camera traps, we subsequently filmed chimpanzees at each of these four TRSs approaching focal trees with a stone in their hand, or grabbing a stone from the base or from inside the tree’s hollow cavities, and then proceeding to throw it (N = 64 total stone throwing events; Table 1). We observed three particular variants of the behaviour: the rock was thrown at the tree using one or both hands (‘hurl’); hit repeatedly against the tree while the chimpanzee held it (‘bang’); or thrown into the hollow tree or a hollow groove formed by large buttress roots (‘toss’; Table 1; Supplementary Movies 1–7). The individuals observed in the camera trap footage were mainly adult males, but we also observed an adult female and a juvenile exhibiting the behaviour (Supplementary Movies 1 and 6). Common to all accumulative stone throwing observations exhibited by adults (N = 63) was the pant hoot vocalization, in particular the introduction and/or build-up phase^25^, which occurred after or while the individual picked up and handled the rock (Fig. 3). The pant hoot is a characteristic feature of the ritualized agonistic displays of adult male chimpanzees, which typically also involves piloerection, bipedal stance, hand and feet drumming on buttress roots of trees and, in some populations, is preceded by leaf-clipping^25^,^26^,^27^. Unfortunately, audio was recorded for only 50 of the 64 events captured on camera traps, so we may underestimate the variation in vocal behaviour accompanying accumulative chimpanzee stone throwing. We further observed that rock handling and throwing was sometimes accompanied by the individual swaying back and forth while bipedal and piloerect, and even leaf-clipping (Supplementary Movie 4; Fig. 3), all behaviours associated with a typical chimpanzee display^27^. When the rock was thrown, this was often, but not always, accompanied by the climax phase of the pant hoot consisting of scream elements and drumming with the hands or feet on the tree^25^,^26^. In some cases we do not have footage of the full series of behaviours since camera trap videos are limited in length (60 seconds), and cameras were triggered at varying times for each accumulative stone throwing event captured.

The accumulative stone throwing behaviour was only observed in Western chimpanzees, *Pan troglodytes verus* (Fig. 1). Moreover, to our knowledge, the behaviour has not been observed at any of the 17 existing mid- to long-term chimpanzee research sites (LRS) across Africa^20^ (Fig. 1). The wear observed on the trunks and buttress roots of trees targeted by the accumulative stone throwing indicated that all of the active sites had been in use for some time. Stone tools appeared to be regularly reused: in 57 of 64 stone throws filmed, the individual picked up a rock from the base of the tree, and once from inside the tree. We also observed the same individual at the same tree repeatedly engaging in accumulative stone throwing (Table 1), suggesting individuals frequently revisit sites.

### Raw material accumulation and availability

At all four TRSs where the behaviour was observed, stones had accrued around the base of the tree at each accumulative stone throwing site. In the few cases when rocks were found piled inside the hollows of trees, or nestled between the grooves of buttress roots, we counted between four and 37 stored rocks (Boé (Fig. 3), Comoé GEPRENAF, and Mt. Nimba). The average weight of the individual stones at Boé was 3.6 kg (range: 0.5–17 kg), at Mt. Nimba 2.06 kg (0.2–7.1 kg) and at Comoé GEPRENAF 0.98 kg (0.8–7 kg).

In order to determine if inter-site variation in the presence or absence of accumulative stone throwing could be explained by ecological factors, we examined the availability of stones and the availability of hollow trees. Using habitat plots and strip transects at 11 West African TRSs, including the four TRSs exhibiting accumulative stone throwing (Table 1), we calculated the density of hollow trees, or trees with hollow grooves between roots, and found no significant difference in hollow tree density between TRSs where accumulative stone throwing had been observed relative to TRSs where it had not been observed (Mann Whitney U test: N_present_ = 3, N_absent_ = 4, U = 4, P = 0.63). While hollow tree density was quite high at Boé, other TRSs also exhibited a high hollow tree density but did not reveal evidence for accumulative stone throwing (e.g., Bakoun and Djouroutou, Table 1). Similarly, a high availability of stones did not appear to be a necessary determinant for the behaviour. The densities of loose rocks calculated along strip transects at each TRS (Table 1), indicated that although the accumulative stone throwing behaviour occurred in habitats with relatively high stone availability, this factor did not guarantee the occurrence of the behaviour. Furthermore, we found no significant difference in rock density between West African TRSs (Table 1), where the behaviour was observed compared with West African TRSs where the behaviour was not observed (N_present_ = 4, N_absent_ = 7, U = 5, P = 0.11). Moreover, no observations of accumulative stone throwing were made at Bakoun, Sobeya or Kayan, although these TRSs also exhibited relatively high rock densities.

## Discussion

The accumulative stone throwing behaviour described here seems to be rare in chimpanzees as it has not been observed during decades of research at long-term field sites across Africa and has not been observed at other PanAf sites (Fig. 1; Supplementary Table 1). This suggests that these initial observations may underestimate the behavioural complexity characteristic of accumulative stone throwing in chimpanzees. The habitual incorporation of stone tools into the ritualized display of chimpanzees is a novel discovery, and according to our data, may be a socially-learned cultural tradition found in limited populations of West African chimpanzees. It remains to be tested whether fine-scale genetic differences may influence the observed distribution of stone tool-use behaviours in this subspecies. However, a genetic explanation seems unlikely to account for the patchy distribution of this behaviour within West Africa (see Fig. 1). We expect that the concentrated accumulation of stone tools at specific tree locations will facilitate further study of this behaviour, for example through determining site fidelity using excavations and other archaeological methods. If sites are found to be long-lived, it would represent another type of stone tool-use behaviour in chimpanzees, in addition to nut-cracking, that leaves behind an archaeological record^8^,^9^.

Our observations raise numerous questions about the potential interpretations of this behaviour. There are at least two contrasting hypotheses that need to be evaluated with long-term studies to elucidate the broader context in which this behaviour is shown. First, the accumulative stone throwing behaviour could be a modification of the male chimpanzee display, since the action bears a close resemblance to hand and feet drumming^26^, a ritualized behaviour found in all known chimpanzee populations. The incorporation of stone tools into this display may serve to enhance the sound propagation properties in more open savannah-woodland landscapes. In this case there should be a clear functional advantage for sound produced by stone throwing compared to hand and feet drumming. For example, fundamental frequencies produced by throwing stones at particular trees may be higher and would thus travel further in a more open environment^28^. Thus, one may hypothesize that stone throwing, specifically the ‘bang’ variant of the behaviour, initially emerged as a variant of hand and feet drumming. The continuous practice of stone throwing and subsequent collection of artefacts at specific trees then led to modification of stone tool availability at these selected trees. This in turn may have influenced chimpanzee behaviour with a preference for site re-visitation, similar to a local stimulus enhancement mechanism proposed for stone-handling behaviour in Japanese macaques^17^. Over time chimpanzee accumulative stone throwing would therefore have become more and more independent of its original trigger (hand and feet drumming) and, as some of our recordings suggest, is now occasionally practiced independently of it (e.g. Supplementary Movies 2 and 4). As such, the accumulation of stones at particular trees may have originated as a by-product of modified display behaviours occurring at fixed locations.

Alternatively, chimpanzee accumulative stone throwing could have emerged from a motivation other than the enhancement of ritualized male displays. In this case one would not necessarily expect a major functional advantage of sound propagation compared to hand and feet drumming. If this were true, these rock accumulation sites may need to be considered in a more symbolic context^1^,^29^. Analyses of the behavioural responses of conspecifics, present or nearby, to stone throwing versus hand and feet drumming may provide crucial insight into the meaning of this behaviour. Additionally, the spatial patterning of nesting and home range use in relation to stone accumulation sites may reveal whether they are centrally, peripherally or randomly located in the territory of a chimpanzee community. In fact, marking territorial boundaries and pathways with cairns has been an important practice of many historical human societies^1^. For example, stone accumulation shrines at ‘sacred’ trees are well described for indigenous West African peoples^4^. Superficially, these cairns appear very similar to what has been described here for chimpanzee accumulative stone throwing sites, thus it would be interesting to explore whether there are any parallels between chimpanzee accumulative stone throwing and human cairn building, especially in regions of West Africa where the local environment is similar.

Incidentally, chimpanzee accumulative stone throwing and the subsequent aggregation of tools at particular trees shares two important features with human ritual practices: the strong association to a particular location or site with a collection of artefacts over time, and ritualized behaviour patterns^30^,^31^. Although there is no overarching, agreed-upon definition of ritual^30^, similarities between ritualized animal behaviour and the repeated, stereotyped behaviours commonly observed during human rituals have already been proposed by anthropologists^31^ and ethologists^32^ as having a common origin, as well as sharing similar neurological pathways^30^,^33^,^34^. From a phylogenetic perspective, we suggest that further research on naturally occurring stone tool-use traditions in nonhuman primates, especially those whose occurrence appears to be tied to specific locations with no immediate functional benefit to the individual, namely accumulative stone throwing in chimpanzees and stone-handling behaviour in Japanese macaques, could serve to enlighten our understanding of the origins of ritual sites. At the very least, stone accumulation sites produced by extant nonhuman primates have the potential to challenge and refine our interpretations of hominin archaeological assemblages in similar contexts^5^,^6^,^8^.

## Methods

We sampled 29 lesser-known chimpanzee communities as part of the Pan African Programme: The Cultured Chimpanzee (PanAf)^23^ to complement the published data already available from 17 mid- to long-term chimpanzee research sites. PanAf data collection was conducted for 14–17 months at 26 TRSs with on-going data collection at an additional three TRSs (ranging from 4–10 months duration; Supplementary Table 1). A further five PanAf TRSs were carried out at established long-term research sites (LRS) on unhabituated chimpanzee communities, also for a 14–17 months duration. At each PanAf TRS a broad spectrum of non-invasive sampling methods was applied following a uniform sampling protocol^24^. One TRS in West Africa, Grebo in Liberia, was in the final months of completion when the discovery of accumulative stone throwing behaviour was made; therefore, stone and hollow tree density were not available for this site.

Stone banging was first heard in Boé, Guinea-Bissau in 2010 by an independent team of field workers. The first confirmed observation of the accumulative stone throwing behaviour occurred by chance at Sangaredi in Guinea in March 2011 after discovering unusual markings on a hollow tree. When investigating the area further, we found conspicuous accumulations of rocks next to trees with clear indications of wear. The subsequent placement of remote video camera traps (Bushnell Trophy Cameras) at these locations revealed the chimpanzees’ accumulative stone throwing behaviour. Following this observation we systematically collected data on this behaviour across all 34 PanAf TRSs. For this we used both recce methodology and opportunistic sampling to try to locate conspicuous accumulation of stones in close proximity to trees. At each TRS we sampled 15–44 km of recces or line transects (Table 1), and transects were walked three times during a one-year period, during which any signs of stone throwing and piling behaviour were sought. In case such locations were found, we then placed one or more video camera traps at a distance of a few metres from the target trees and stone piles. Video cameras were strapped to trees at a height of 1 m and were operational 24 hours per day. When rocks were found inside hollow trees we counted and weighed them using a spring scale (35 kg Travel Blue Spring scale). We extracted the following information from each video that contained observations of chimpanzee accumulative stone throwing: time, date, number of individuals present, age and sex class for each individual, and type of behaviour exhibited. The accumulative stone throwing behaviour was classified according to the type of rock handling observed: ‘hurl’, ‘bang’ or ‘toss’, and we also noted the occurrence of other behaviours such as pant hooting and elements of the ritualized chimpanzee display (Fig. 3).

The density of hollow trees was calculated either by observations of these trees while conducting strip transects, or from 20 m by 20 m habitat plots that were surveyed at each TRS (208–246 habitat plots per TRS). Habitat plots were centered along transects at 100 m intervals. Stone availability was estimated using strip transect methodology, whereby observers count the number of loose rocks found within 1 m of either side of the line transect or recce being walked. A detailed protocol for all PanAf methods is freely available online on our website http://panafrican.eva.mpg.de.

### Ethics statement

The data collection methods for this study were strictly non-invasive and were approved by the Ethical Board of the Max Planck Society. As such, the study was conducted in accordance with Germany’s laws and the rules and regulations governing animal research in the European Union. In every African country where field data were collected, all relevant permissions were first obtained from the country’s governmental institutions before starting data collection.

## Additional Information

**How to cite this article**: Kühl, H. S. *et al.* Chimpanzee accumulative stone throwing. *Sci. Rep.* **6**, 22219; doi: 10.1038/srep22219 (2016).

## Supplementary Material

Supplementary Information

Supplementary Movie 1

Supplementary Movie 2

Supplementary Movie 3

Supplementary Movie 4

Supplementary Movie 5

Supplementary Movie 6

Supplementary Movie 7

## Figures and Tables

**Table 1:** Data from 11 temporary research sites (TRSs) across West Africa where data were collected for 14–17 months between 2011 and 2014 to document the occurrence of chimpanzee accumulative stone throwing.

	TRS	Long	Lat	Size (km^2^)	Transect length (km)	Rock density (/km^2^)	Hollow tree density (/km^2^)	# of stone throws	# sites (hollow trees)	# sites behaviour recorded (hollow trees)	# of individuals	# of revisits at a site^1^	Age-sex class	Stone throw variant
Côte d’Ivoire	Djouroutou	–7.28	5.37	35	35.0	1500	120.5*	–	–	–	–	–	–	–
	Comoé GEPRENAF	–3.71	8.84	69	39.0	131,244	528.6	23	9 (2)	3(2)	4–6	1–6	AM	H, B, T
	Taï R	–7.33	5.87	25	25.0	1640	103*		–	–	–	–	–	–
	Taï E	–7.31	5.89	25	30.0	350	86*		–	–	–	–	–	–
Guinea	Bakoun	–12.5	11.9	48	40.0	59,325	700		–	–	–	–	–	–
	Sangaredi	–13.77	11.1	91	24.0	24,229	NA	3	10 (2)	2(2)	3	0	AM, J	H, B/T§
	Sobeya	–11.71	10.26	96	27.0	81,121	NA		–	–	–	–	–	–
Guinea Bissau	Boé	–14.22	11.75	56	43.8	99,189	251.1*	33	28 (2)	6(2)	10–12	0–3	AM, AF	H, T
Liberia	Mt. Nimba	–8.49	7.22	25	25.0	520,800	120	5	14 (1)	2(0)	2	1–3	AM	H
	Sapo	–8.41	5.41	20	15.0	0	NA		–	–	–	–	–	–
Senegal	Kayan	–12.29	13.18	75	29.0	308,603	NA		–	–	–	–	–	–

Size refers to data collection area. #of individuals refers to number of unique individuals observed to perform the behaviour. For cases where identity could not be confirmed we state a range. AM-adult male, J-juvenile, AF-adult female. H-hurl, B-bang, T-toss variant of accumulative stone throwing behaviour.

^1^Only revisits by individuals that could be clearly identified are included; therefore this is likely to be an underestimate.

^*^Data from habitat plots not transects (see Methods).

^§^Observation of a juvenile (see also Supplementary Movie 6).
