# Supplementary material for: Chimpanzee accumulative stone throwing
Source: Sci Rep. 2016 Feb 29;6:22219. doi: 10.1038/srep22219 (PMC4770594; doi:10.1038/srep22219)
Supplement: Supplementary Information [file srep22219-s1.pdf]

Chimpanzee accumulative stone throwing

Hjalmar S. Kühl[1,2], Ammie K. Kalan[1*], Mimi Arandjelovic[1], Floris Aubert[3], Lucy D'Auvergne[3], Annemarie Goedmakers[4], Sorrel Jones[1], Laura Kehoe[3], Sebastien Regnaut[3], Alexander Tickle[1], Els Ton[1,4], Joost van Schijndel[1,4], Ekwoge E. Abwe[5], Samuel Angedakin[1], Anthony Agbor[1], Emmanuel Ayuk Ayimisin[1] , Emma Bailey[1], Mattia Bessone[1], Matthieu Bonnet[6], Gregory Brazolla[1], Valentine Ebua Buh[1], Rebecca Chancellor[7], Chloe Cipoletta[8], Heather Cohen[1], Katherine Corogenes[1], Charlotte Coupland[1], Bryan Curran[6], Tobias Deschner[1], Karsten Dierks[1], Paula Dieguez[1], Emmanuel Dilambaka[8], Orume Diotoh[9], Dervla Dowd[3], Andrew Dunn[8], Henk Eshuis[1], Rumen Fernandez[1], Yisa Ginath[1], John Hart[10], Martijn Ter Heegde[11], Thurston Cleveland Hicks[1], Inaoyom Imong[1,8], Kathryn J. Jeffery[12,13,14], Jessica Junker[1], Parag Kadam[15], Mohamed Kambi[1], Ivonne Kienast[1], Deo Kujirakwinja[8], Kevin Langergraber[16], Vincent Lapeyre[3], Juan Lapuente[1], Kevin Lee[1], Vera Leinert[3], Amelia Meier[1], Giovanna Maretti[1] , Sergio Marrocoli[1], Tanyi Julius Mbi[1], Vianet Mihindou[12], Yasmin Moebius[1], David Morgan[8,17], Bethan Morgan[5,18], Felix Mulindahabi[8], Mizuki Murai[1], Protais Niyigabae[8], Emma Normand[3], Nicola Ntare[8,] Lucy Jayne Ormsby[1], Alex Piel[19], Jill Pruetz[20], Aaron Rundus[21], Crickette Sanz[8,22], Volker Sommer[23], Fiona Stewart[15], Nikki Tagg[24], Hilde VanLeeuwe[8], Virginie Vergnes[3], Jacob Willie[24], Roman M. Wittig[1,25], Klaus Zuberbuehler[26], Christophe Boesch[1,3]

Affiliations

[1] Max Planck Institute for Evolutionary Anthropology (MPI EVAN), Deutscher Platz 6, 04103 Leipzig
[2] German Centre for Integrative Biodiversity Research (iDiv) Halle-Leipzig-Jena, Deutscher Platz 5e, 04103 Leipzig
[3] Wild Chimpanzee Foundation (WCF), Deutscher Platz 6, 04103 Leipzig
[4] Chimbo Foundation, Amstel 49, 1011 PW Amsterdam, Netherlands
[5] Ebo Forest Research Project, BP3055, Messa, Cameroon
[6] The Aspinall Foundation, Port Lympne Wild Animal Park, Hythe, Kent, UK
[7] West Chester University, Departments of Anthropology & Sociology and Psychology, West Chester, PA, USA
[8] Wildlife Conservation Society (WCS), 2300 Southern Boulevard. Bronx, New York 10460, USA
[9] Korup Rainforest Conservation Society, c/o Korup National Park, P.O. Box 36 Mundemba, South West Region, Cameroon
[10] Lukuru Foundation, 1235 Avenue des Poids Lourds / Quartier de Kingabois, Kinshasa, DRC
[11] WWF Cameroon Country Office, BP6776; Yaoundé, Cameroon
[12] Agence National des Parcs Nationaux (ANPN) Batterie 4, BP20379, Libreville, Gabon
[13] School of Natural Sciences, University of Stirling, UK
[14] Institute de Recherche en Ecologie Tropicale, Libreville, Gabon
[15] University of Cambridge, Pembroke Street, Cambridge, UK CB2 3QG
[16] Arizona State University, PO Box 872402, Tempe, AZ 85287-2402 USA
[17] Lester E. Fisher Center for the Study and Conservation of Apes, Lincoln Park Zoo, 2001 North Clark Street, Chicago, Illinois 60614 USA
[18] Institute for Conservation Research, Zoological Society of San Diego, Escondido, CA 92025, USA
[19] School of Natural Sciences and Psychology, Liverpool John Moores University, James Parsons Building, Rm653 Byrom Street, Liverpool L3 3AF, UK
[20] Iowa State University, Department of Anthropology, 324 Curtiss Hall, Ames, IA 50011, USA
[21] West Chester University, Department of Psychology, 700 S High St., West Chester, PA, 19382 USA
[22] Washington University Saint Louis, Department of Anthropology, One Brookings Drive, St. Louis, MO 63130, USA
[23] University College London, Department of Anthropology, 14 Taviton Street, London WC1H 0BW, UK
[24] KMDA, Centre for Research and Conservation, Royal Zoological Society of Antwerp, Koningin Astridplein 20-26, B-2018 Antwerp, Belgium
[25] Taï Chimpanzee Project, Centre Suisse de Recherches Scientifiques, BP 1301, Abidjan 01, CI
[26] Université de Neuchâtel, Institut de Biologie, Rue Emile-Argand 11, 2000 Neuchâtel, Switzerland

Corresponding author: *Ammie K. Kalan, ammie_kalan@eva.mpg.de

Supplementary Table 1. Site name and coordinates of all PanAf temporary research sites (TRSs) where data on chimpanzee accumulative stone throwing behaviour were collected or on-going, and all mid- to long-term research sites (LRS) on habituated chimpanzees where the behaviour is absent from published literature (see Figure 1 in the main manuscript). TRS at LRS refers to sites where the PanAf data collection protocol was carried out at a TRS on the LRS chimpanzee study community. PanAf TRS duration was 14-17 months unless otherwise stated.

Country	Site	Longitude	Latitude	Site
**Cameroon**	Campo Ma'an	10.463100	2.637160	TRS
**Cameroon**	Ebo	10.330000	4.319000	LRS
**Cameroon**	Ebo southwest	10.383333	4.600000	TRS
**Cameroon**	Korup	9.057099	5.286061	TRS
**Cameroon**	La Belgique/Dja	13.150000	3.416670	TRS at LRS
**Cameroon**	Mt Cameroon	9.172730	4.217340	TRS
**Côte d'Ivoire**	Comoé GEPRENAF	-3.707890	8.841651	TRS
**Côte d'Ivoire**	Djouroutou	-7.283333	5.366667	TRS
**Côte d'Ivoire**	Mt Sangbe	-7.262975	7.952841	TRS
**Côte d'Ivoire**	Taï E	-7.314150	5.893359	TRS
**Côte d'Ivoire**	Tai east	-7.3333	5.8667	LRS
**Côte d'Ivoire**	Tai middle	-7.3333	5.8667	LRS
**Côte d'Ivoire**	Tai north	-7.3333	5.8667	LRS
**Côte d'Ivoire**	Taï R	-7.333300	5.866700	TRS
**Côte d'Ivoire**	Tai south	-7.3333	5.8667	LRS
**DR-Congo**	Bili-Uere	25.868755	4.602616	TRS
**DR-Congo**	Kabogo[1]	29.694487	-7.129425	TRS
**DR-Congo**	Kahuzi Biega	28.726501	-2.155465	LRS
**DR-Congo**	Rubi-Tele[2]	24.856460	2.647449	TRS
**Gabon**	Bateke	14.041160	-2.167566	TRS
**Gabon**	Loango	9.480000	-2.090000	TRS at LRS
**Gabon**	Lope	11.583300	-0.166700	TRS
**Guinea**	Bakoun	-12.500000	11.900000	TRS
**Guinea**	Bossou	-8.5000	7.6500	LRS
**Guinea**	Sangaredi	-13.766667	11.100000	TRS
**Guinea**	Sobeya	-11.709126	10.259386	TRS
**Guinea Bissau**	Boe	-14.216666	11.750000	TRS
**Liberia**	Grebo	-7.732278	5.405889	TRS
**Liberia**	Mt. Nimba	-8.493388	7.219052	TRS
**Liberia**	Sapo	-8.414625	5.411114	TRS

**Mali**	Bafing[3]	-10.307343	12.578020	TRS
**Nigeria**	Gashaka	11.700000	7.566667	TRS at LRS
**Nigeria**	Mbe	9.041748	6.257967	TRS
**Republic of Congo**	Conkouati	11.416667	-3.800000	TRS
**Republic of Congo**	Goualougo	16.866667	2.100000	LRS
**Rwanda**	Gishwati	29.427028	-1.747220	TRS at LRS
**Rwanda**	Nyungwe	29.292778	-2.490000	TRS
**Senegal**	Fongoli	-12.166	12.666	LRS
**Senegal**	Kayan	-12.293810	13.182780	TRS
**Senegal**	Mt. Assirik	-12.7667	12.8833	LRS
**Tanzania**	Gombe	29.61643	-4.695511	LRS
**Tanzania**	Mahale	29.7333	-6.1167	LRS
**Tanzania**	Semliki	30.3333	0.8333	LRS
**Tanzania**	Ugalla	30.616700	-5.316700	TRS at LRS
**Uganda**	Budongo	31.500000	1.750000	LRS
**Uganda**	Budongo east	31.500000	1.750000	TRS
**Uganda**	Bwindi	29.708330	-1.008333	TRS
**Uganda**	Kalinzu	30.117	-0.283	LRS
**Uganda**	Kanyawara	30.3667	0.5667	LRS
**Uganda**	Ngogo	30.432130	0.546562	LRS
**Uganda**	Ngogo east	30.462340	0.523217	TRS

[1]Duration of site 4 months
[2]Duration of site 10 months
[3]Duration of site 7 months

Supplementary Movie Data

Supplementary Movie 1. Adult female chimpanzee picks up a rock and hurls it at the tree at Boé, Guinea-Bissau.

Supplementary Movie 2. Adult male chimpanzee takes a rock from the hollow of a tree and hurls it again at the same tree at Boé, Guinea-Bissau.

Supplementary Movie 3. Adult male chimpanzee tosses a stone into the hollow cavity of a tree at Boé, Guinea-Bissau.

Supplementary Movie 4. Adult male chimpanzee at Nimba, Liberia leaf clips, then picks up a rock and hurls it at the tree.

Supplementary Movie 5. A one-armed adult male chimpanzee hurls a rock at a tree at Nimba, Liberia.

Supplementary Movie 6. A juvenile chimpanzee bangs a stone against a tree at Sangaredi, Guinea before tossing it into the tree cavity.

Supplementary Movie 7. An adult male chimpanzee picks up a rock and bangs it against the tree at GEPRENAF, Côte d'Ivoire.
